# Age-Related Social Selection and Its Associated Emotional and Cognitive Costs Across Adulthood

**DOI:** 10.1093/geroni/igaa057.1268

**Published:** 2020-12-16

**Authors:** Hsiao-Wen Liao, Laura Carstensen

**Affiliations:** 1 Stanford University, Palo Alto, California, United States; 2 Stanford University, Stanford, California, United States

## Abstract

Socioemotional selectivity theory maintains that goal prioritization differs across adulthood as a function of future time horizons. To prepare for a long and nebulous future, young adults prioritize learning and exploration over emotional meaning. Relieved from the burden to prepare, older adults prioritize emotionally meaningful goals. In the context of social relationships, younger adults include proportionally fewer familiar social partners in their social networks, whereas older adults’ social networks encompass proportionally fewer unfamiliar social partners. Although social selection is considered adaptive, it inevitably involves gains and losses. The current study examined whether age-related selectivity correlates with (1) greater concurrent negative emotions in younger people, and (2) poorer cognitive performance in old age. A life-span sample (N = 258) completed a social networks questionnaire and cognitive tests. Daily emotional experience was assessed using experience sampling. A subset (N = 119) completed the cognitive tests again five years later. Results of multiple regression analysis, controlling for physical health and trait neuroticism, indicate that smaller proportions of familiar social partners in one’s social network correlated with more frequent experience of negative emotions. Age moderated this association with a stronger association in younger than older people. Results of separate multiple regression analysis, controlling for baseline cognition, physical health, age, SES, and trait openness, indicate that a smaller proportion of social partners in one’s outer social circle negatively predicted older adults’ Digit Span Backward performance assessed five years later. We discuss our findings within the framework of gains and losses in life-span development.

